# Identification of transdiagnostic phenomena among patients, the general population, relatives, and mental health professionals using topic modeling techniques

**DOI:** 10.3389/fpsyg.2025.1715108

**Published:** 2026-01-06

**Authors:** Alexis Vancappel, Hugo Kazzi, Hayfa Zgaya-Biau, Rodolphe Saur, Eva Fourel, Robert Courtois, Géraldine Tapia, Thierry Kosinski, Arnaud Carré, Catherine Bortolon, Fanny Marteau-Chasserieau, Lucia Romo, Celine Baeyens, Yannick Morvan, Chrystel Besche-Richard, Wissam El-Hage

**Affiliations:** 1 Univ. Lille, ULR 4072 - PSITEC - Psychologie: Interactions Temps Émotions Cognition, Lille, France; 2 Univ. Lille, UMR 9189 - CRIStAL - Centre de Recherche en Informatique Signal et Automatique de Lille, Lille, France; 3 Laboratoire AxiomaVox, Paris, France; 4 Department of Psychology, QualiPsy, University of Tours, Tours, France; 5 Univ. Bordeaux, LabPsy, UR, Bordeaux, France; 6 Univ. Grenoble Alpes, Univ. Savoie Mont Blanc, LIP/PC2S, Grenoble, France; 7 Institut Universitaire de France, IUF, Paris, France; 8 Équipe VCR École de Psychologues Praticiens de l’Institut Catholique de Paris, UR Religion, Culture et Société, EA, Paris, France; 9 Université Paris Nanterre, UR 4430 CLIPSYD, Hopital Universitaire Raymond-Poicaré (APHP), Inserm CESP, Nanterre, France; 10 Université de Reims Champagne-Ardenne, C2S, Reims, France; 11 Groupe Hospitalier Paul Guiraud, GHT Psy Sud, Boulogne-Billancourt, France; 12 CHRU de Tours, Pôle de Psychiatrie-Addictologie, Centre Régional de Psychotraumatologie CVL, UMR, iBrain, Université de Tours, Inserm, Tours, France

**Keywords:** bidirectional encoder representations from transformers, cognitive behavioral therapy, contextual model, model languages, psychiatry, psychopathology, topic modeling, transdiagnostic approach

## Abstract

**Introduction:**

Recent research has highlighted the limitations of the categorical approach to mental disorders and has increasingly supported the development of a transdiagnostic perspective. This emerging approach focuses on common distal factors (circumstantial, biological, and social) and psychological processes that contribute to psychological suffering across a range of disorders, as well as on the resulting psychological symptoms. The present study aims to identify transdiagnostic distal factors, psychological processes, and symptoms by analyzing narratives through topic modeling—an unsupervised machine learning technique, specifically within Natural Language Processing (NLP). Topic modeling enables the automatic extraction of latent themes from unstructured text, making it possible to identify psychological patterns grounded in patients’ lived experiences.

**Methods:**

We recruited four groups of participants: Patients diagnosed with a psychiatric disorder (*N* = 445), Individuals from the general population (*N* = 570), Relatives of patients with psychiatric disorders (*N* = 354), and Mental health professionals (*N* = 131). Participants answered open-ended questions exploring the causes of psychological suffering, their wishes for change, and their previous experiences with psychotherapy.

**Results:**

We identified 258 topics, which were organized into 12 overarching themes. The most prominent topics concerned *Emotional and Psychological Difficulties*, *Family and Social Relationships*, and *Therapeutic Processes*. Each theme showed a comparable prevalence across the different diagnostic categories, supporting the transdiagnostic nature of these phenomena.

**Conclusion:**

Topic modeling can be used effectively to identify transdiagnostic distal factors, psychological processes, and symptoms from diverse narratives. This approach tends to provide a novel means of supporting the relevance and validity of the transdiagnostic perspective.

## Introduction

Mental health disorders are widespread and represent a significant public health issue. To address these challenges, researchers and academic societies have primarily adopted a categorical approach, aimed at determining the presence or absence of various disorders, based on clearly defined sets of criteria (Widakowich et al., 2013) as in the International Classification of Diseases (ICD) (World Health Organization, 2019) and the Diagnostic and Statistical Manual of Mental Disorders (DSM; American Psychiatric Association, 2022). Accordingly, in clinical settings, practitioners are encouraged to identify patients’ diagnoses and to propose appropriate treatments based on this diagnostic evaluation. In this context, numerous psychotherapy treatment guidelines have been developed for specific disorders (American Psychiatric Association, 2025; Haute autorité de Santé, 2009, 2017; Phoenix Australia, 2021). This approach appears largely relevant, and psychotherapy has been shown to be effective when applied within this framework (Barlow, 2004).

However, several limitations of the diagnostic approach have recently been highlighted (Dalgleish et al., 2020). The categorical approach assumes that each disorder has an essential, underlying nature that causes its manifestation (der Van Linden, 2016). Yet, no such essence has been identified to date, and, similarly, no disorder-specific biological markers have been found (Lakhan et al., 2010). Moreover, patients often present non-specific symptoms that are not fully explained by a single diagnosis (Zimmerman et al., 2000), and comorbidity between multiple concurrent disorders is a frequently occurring phenomena (Brown et al., 2001).

**Figure 1:**
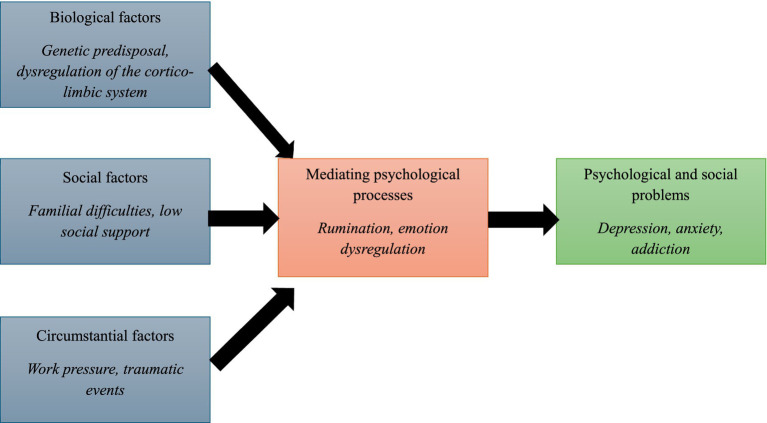
Kinderman’s model (Kinderman, 2005).

These limitations have contributed to the emergence of the transdiagnostic approach, exemplified by Kinderman (2005), as illustrated in Figure 1. According to this model, biological factors (e.g., reduced gray matter volume), social factors (e.g., low social support), and circumstantial factors (e.g., stressful life events) influence mediating psychological processes (e.g., emotion regulation abilities), which in turn lead to psychological symptoms (e.g., depression, hallucinations). Within this framework, the emphasis is placed on psychological processes as central and proximal mechanisms involved in the development and maintenance of psychiatric disorders. Therefore, Kinderman (2005) distinguishes between two levels of psychological phenomena.

The first is symptoms, and the second is psychological processes. Psychological processes are thought to play a causal role in psychological difficulties, whereas symptoms are considered the outcomes of such phenomena. For instance, Kinderman (2005, p. 17) states that *“hallucination is seen as a product of a psychological act—the misattribution of the origin of a percept.”* Here, hallucination is the symptom, and misattribution is the process.

Harvey (2004) define a psychological process as “an aspect of cognition (attention, memory, reasoning, thinking) or behavior (e.g., avoidance) that may contribute to the maintenance of a psychological disorder.”

Building on these frameworks, several researchers have attempted to identify key psychological processes. For instance, the Research Domain Criteria (RDoC) (Cuthbert, 2021) propose five broad categories of processes that may explain psychopathological mechanisms. These domains are: (1) Negative Valence Systems (e.g., fear, anxiety, and loss); (2) Positive Valence Systems (e.g., reward responsiveness, reward learning, and motivation); (3) Cognitive Systems (e.g., attention, perception, and working memory); (4) Social Processes (e.g., affiliation and attachment, social communication, and understanding of others); and (5) Arousal and Regulatory Systems (e.g., arousal, circadian rhythms, and sleep difficulties).

More recently, Vancappel et al. (2024) recommended focusing on transdiagnostic psychological skills as potential targets for psychotherapy. They identified seven core skills commonly implicated in psychiatric disorders and developed a transdiagnostic skills-training program to treat patients accordingly (Vancappel et al., 2025): (1) Emotion regulation; (2) Behavioral activation and planning; (3) Emotional identification; (4) Assertiveness; (5) Problem-solving; (6) Emotional confrontation; and (7) Control of cravings and urges.

Another research group (Wade et al., 2025) recently proposed a broader list of 38 psychological processes (e.g., sleep problems, anxiety sensitivity) involved in anxiety, depression, and eating disorders, based on a comprehensive literature review.

Altogether, this body of work highlights the challenge of establishing an exhaustive and clinically useful list of psychological processes.

On the one hand, relying solely on the empirical conceptualizations of a single research team (such as Vancappel) may lead to the omission of important processes.

On the other hand, integrating multiple conceptualizations risks producing an overly extensive and redundant list, where similar concepts are labeled differently.

Therefore, new Artificial Intelligence (AI)–based techniques offer promising perspectives for reducing redundancy between concepts. In particular, Natural Language Processing (NLP) analyzes how people use vocabulary to detect potentially redundant concepts. Such techniques calculate the interrelations between words or semantic units (tokens). Tokens that are similarly related to the same other tokens are assumed to overlap and to represent a similar concept. For instance, if *“anxiety”* and *“stress”* are used interchangeably in everyday language, NLP can detect that these two tokens constitute a similar concept in participants’ minds. Moreover, the exploration of transdiagnostic symptoms and distal factors (circumstantial, social, and biological), as proposed by Kinderman (2005), has received relatively little attention and could benefit from the use of AI-based techniques.

AI-based techniques have been increasingly used to address various issues in mental health. AI has primarily been applied to develop algorithms that detect psychiatric disorders and improve early diagnosis. A recent literature review and bibliometric mapping highlighted the growing interest in AI-based methods, particularly machine learning and deep learning, for the detection of psychiatric disorders. This work identified that such research is conducted mainly in the United States and China, with a strong focus on detecting schizophrenia and depression (Sharma and Chariar, 2024).

These studies rely on various databases to train algorithms. For instance, Sharma et al. (2025) used speech and behavioral data, achieving a competitive accuracy of 99.06% in distinguishing between normal and pathological conditions. Similarly, Choudhury et al. (2026) used electroencephalogram (EEG) data to evaluate the presence of Major Depressive Disorder, reaching a binary classification accuracy of up to 99.22%. However, no research has been conducted to detect psychological processes using a transdiagnostic approach.

Therefore, the aim of the present study was to explore the phenomena that may be involved in psychopathology, drawing on experiential knowledge rather than purely theoretical models. To achieve this, we followed the general conceptual organization proposed by Kinderman (2005), exploring symptoms, psychological process, and distal factors (biological, circumstantial and social). We integrated insights from patients, members of the general population, relatives, and clinicians to develop a broader and more grounded understanding of the psychological symptoms and processes and distal factors implicated in psychiatric disorders. These four groups were chosen because they offer complementary perspectives: patients provide first-hand accounts of lived experience, relatives contribute insights into interpersonal dynamics, clinicians bring professional expertise, and members of the general population offer a normative reference point. This diversity enhances the ecological validity and robustness of the processes identified. A second aim of this paper was to strengthen the idea that similar symptoms, processes and distal factors are involved across different diagnoses, using new data-analysis and natural language processing tools.

## Methods

### Participants

We recruited four samples of participants. The first sample consisted of outpatients from the psychiatric services of the University Hospital Center of (blinded) (*N* = 445; mean aged = 34.88 years; standard deviation = 11.91). This dataset was retrospective, as we used responses to three open-ended questions that patients answered when requesting their first appointment with a clinician. The second sample was composed of individuals from the general population (*N* = 570; mean aged = 33.5 years; standard deviation = 13.3). The third consisted of relatives of people with mental health issues (*N* = 354; mean aged = 35.5 years; standard deviation = 18.32), and the fourth of mental health professionals (*N* = 131; mean aged = 36.6 years; standard deviation = 10). For these second and third samples, data were collected online via both social media and a panelist recruitment platform. Panelists represented 53.68% for the second sample and 89.83% for the third sample. Data from the fourth sample was gathered though social medias and professional mailing lists.

Before participating in the study, all participants in each sample were required to read an information sheet and provide informed consent to take part in the study and share their data. The consent procedure was approved by the ethics committee of both the University Hospital Center and the University of Tours. To be eligible, participants had to be at least 18 years old and fluent in French.

### Measures

Participants were required to provide sociodemographic and professional information, including their gender, age, and occupation. Depending on the sample, additional information was requested, such as the nature of the relationship with the person suffering from a mental health condition (for the third sample), or the clinical setting in which they worked (for the fourth sample). All sociodemographic data are presented in Table 1. For the first sample, we used a broad “other psychiatric disorders” category, as many patients reported multiple comorbidities and highly specific diagnoses (e.g., unspecified dissociative disorder, non-epileptic seizures, adjustment disorder, and so on). We focused on the most common categories to reduce the complexity of the description and to avoid excessively reducing the statistical power of subsequent analyses.

**Table 1:** Demographics information of the four samples.

	N (percentage)			
	Sample 1 clinical population	Sample 2 general population	Sample 3 relatives	Sample 4 professional in mental health
Gender				
Men	159	393	133	27
Women	286	168	219	101
Other	0	9	2	3
Level of education				
Under high school	115	14	33	0
High school	110	45	52	0
One to 3 years of superior education	143	193	98	37
Four or 5 years of superior education	50	260	134	68
More than 5 years of superior education	9	58	37	26
Information not available	16	0	0	0
Professional activity				
Farmer	0	4	1	–
Craftsman	14	9	6	–
Business owner/entrepreneur	0	22	10	–
Executives and higher intellectual professions	25	171	104	–
Intermediate occupation	15	36	32	–
Manual worker	15	12	8	–
Homemaker	0	11	5	–
Employees	163	120	121	–
Student	100	173	57	–
Other	113	12	10	–
Psychiatrist or child psychiatrist	–	–	–	7
Physician	–	–	–	5
Nurse	–	–	–	14
Psychologist	–	–	–	71
Special education worker	–	–	–	1
Sophrologist	–	–	–	2
Other	–	–	–	31
Psychiatric disorders				
Depression	136	81	127	–
Anxiety	222	89	76	–
Bipolar disorder	19	5	36	–
Alcohol use disorder	30	1	9	–
Personality disorder	50	15	16	–
Eating disorder	43	10	6	–
Other psychiatric disorder	231	–	–	–
Psychotherapies performed				
No psychotherapy	–	242	136	–
Psychoanalysis	–	71	54	–
Cognitive behavior therapy	–	104	49	–
Systemic therapy	–	20	9	–
Humanist therapy	–	15	7	–
Supportive therapy	–	71	38	–
Eye movement desensitization and reprocessing	–	71	15	–
Lifespan integration	–	5	1	–
I do not know the name of my therapy	–	121	101	–
Other	–	0	0	–

Overall, patients described in the third sample had a mean age of 41.2 years (SD = 16.8). Of these, 42.94% were men, 55.61% were women, and 1.45% were non-binary. The respondents in this sample were parents (24.86%), siblings (14.97%), romantic partners (36.16%), friends (16.38%), and individuals with another type of relationship (7.63%).

For the clinical sample, three open-ended questions were asked to explore: (1) what caused the patient the most distress, (2) what they would like to change, and (3) why did they ask a consultation. For the three other samples, participants were asked to respond to 10 open-ended questions. These questions explored thoughts, emotions, behaviors, relationships, situations, and other phenomena that may cause psychological distress. They also addressed what participants would like to change in their daily lives, how psychotherapy may have helped them, what aspects of therapy were effective according to them, and what may have hindered progress according to them, when applicable. Participants from the general population were asked to respond based on their own experience (e.g., “What thoughts make you suffer daily?”). In the third sample, respondents answered with regard to their relative, (e.g., “What thoughts make your relative suffer daily?”), and in the fourth sample, mental health professionals were asked to respond based on their experience with patients (e.g., “What thoughts make your patient suffer daily?”).

### Computation and data analysis

The automated analysis of open-ended responses was conducted using the BERTopic method (Grootendorst, 2022), a topic modeling technique developed within the field of Natural Language Processing (NLP). BERTopic combines transformer-based embeddings, dimensionality reduction, and density-based clustering to identify latent semantic themes in textual corpora.

The processing pipeline followed a multi-step structure:

#### Text preprocessing

Responses were first cleaned using an automated Python procedure leveraging the spaCy library for French (fr_core_news_md). Preprocessing steps included: lowercasing, punctuation removal, stopword filtering (via a custom, domain-specific list), and lemmatization (reducing words to their base form).

#### Text vectorization

The preprocessed texts were transformed into dense semantic vectors using the multilingual transformer model distiluse-base-multilingual-cased-v1 from SentenceTransformers. This embedding model is specifically optimized for semantic comparison across languages and is particularly suited for French corpora.

#### Dimensionality reduction (UMAP)

To preserve semantic relationships while reducing computational complexity, the high-dimensional embeddings were projected into a lower-dimensional space using UMAP (Uniform Manifold Approximation and Projection). This step facilitates effective clustering by preserving the global and local structure of the data.

#### Topic clustering (HDBSCAN)

The reduced embeddings were clustered using HDBSCAN, a hierarchical density-based clustering algorithm capable of discovering arbitrarily shaped clusters and identifying outliers. This unsupervised step automatically determined the number of topic groups without requiring it to be predefined. We used several indices to select the most appropriate number of topics. More precisely, we retained the final topic configuration that showed (i) the highest coherence score (C_v), (ii) the highest silhouette score [S(i)], and (iii) the lowest Davies–Bouldin (DB) score. The formulas for these indices are presented above.

#### Coherence measure (cv)

The C_v coherence measure is based on Normalized Pointwise Mutual Information (NPMI) between the words of a topic.

NPMI formula


$$\text{NPMI}(w_{i},w_{j})=\frac{\log\frac{P(w_{i},w_{j})}{P(w_{i})\;P(w_{j})}}{-\log P(w_{i},w_{j})}$$

Topic coherence.

For a topic containing words $w_{1},\ldots ,w_{k}$:


$$C_{v}=\mathrm{Agg}{\left(\text{NPMI}(w_{i},w_{j})\right)}_{1\leq i<j\leq k}$$

where Agg = an aggregation function (typically the average or a weighted average).

#### Silhouette score S(i)

For each point i (e.g., a document embedding):

- a(i)= average distance between i and all other points in the same cluster
- b(i)= average distance between i and all points in the nearest other cluster
  $$s(i)=\frac{b(i)-a(i)}{\max\{a(i),b(i)\}}$$

Higher values (close to 1) indicate dense and well-separated clusters.

#### Davies–Bouldin Index (DB)


$$\mathrm{DB}=\frac{1}{K}\sum_{k=1}^{K}(\max_{j\neq k}\frac{S_{k}+S_{j}}{M_{\mathrm{kj}}})$$

where:

- $S_{k}$= within-cluster dispersion of cluster k
- $M_{\mathrm{kj}}$= distance between the centroids of clusters kand j

Lower DB values indicate better cluster quality (compact and well-separated clusters).

Topic extraction was performed independently for each open-ended question in each sample. This means that a separate topic model was generated for the responses from the general population to the first question (“Which thoughts make you suffer daily?”), then for the responses to the second question (“Which emotion makes you suffer daily?”), and so on until all responses in this sample were processed. A similar procedure was applied to the responses in the other samples until every question and every sample had been fully analyzed.

This approach allowed us to maintain a high level of interpretability, as the answers appeared in different contexts (different questions and different participants) and could not have been meaningfully interpreted if grouped together.

#### Topic representation (c-TF-IDF)

For each identified topic, class-based TF-IDF (c-TF-IDF) was applied to extract the most representative weighted keywords. This representation improves interpretability by highlighting discriminative terms that define each cluster.

#### Post-processing and filtering

Additional filtering was applied to exclude responses that were too short (fewer than three significant words), duplicates, or semantically uninformative (e.g., numeric-only entries). Parameters for the BERTopic model—such as min_topic_size = 5 (minimum number of documents per topic) and n_topics = 10 (maximum number of topics extracted per column)—were used to guide the granularity and relevance of the topic modeling process.

#### Illustration and validation

For each topic, the top 10 weighted keywords were extracted, and two representative verbatim responses were selected to illustrate the theme. To facilitate interpretation, generative AI (GPT-4.0) was used to propose human-readable topic labels and concise summaries based on the keyword sets.

These outputs were then reviewed and refined by the lead researcher to ensure conceptual coherence and domain relevance.

#### Categorization of topics using generative IA

After obtaining the initial list of 258 topics, we classified them into theme and subtheme using GPT-4.0 and the following prompt: “I will provide you with a list of topics that were generated using topic modeling based on the HDBSCAN method and natural language processing techniques. These topics were derived from the narratives of patients, mental health professionals, relatives of patients, and members of the general population. They describe the psychological difficulties people may experience, what they would like to change through therapy, and what they have previously done in the context of past therapeutic interventions. I would like you to categorize these topics by organizing them into themes and subthemes. The themes must be coherent. Each theme and subtheme must include at least two items. You should also minimize the ‘other’ category as much as possible while remaining reasonable and avoiding the creation of unnecessary themes. The final results must include: (1) the theme, (2) the subtheme, and (3) the original topic. They should be presented in English in an Excel or CSV table.” This procedure allowed us to obtain the table presented in Supplementary material 1.

#### Labialization of the themes and subthemes

Finally, the researchers classified the output as related to psychological symptoms, psychological processes, or distal factors, following Kinderman (2005) model and definitions. The first author made the initial proposal, and the classification was approved by all other researchers without requiring any modifications, as it was clear and unanimously agreed upon.

#### Cross-analysis with diagnoses

Finally, the extracted topics were cross-referenced with participants’ reported psychiatric diagnoses to explore theme prevalence across clinical subgroups. We used repeated-measures ANOVA to compare the degree of affiliation for each topic. In this analysis, the unit of analysis was the topic itself (one topic corresponding to one participant). The independent variables were the general theme (between-subjects variable) and the different diagnoses (within-subjects variable).

We assessed the overall effect size within the ANOVA using η^2^ (Cohen, 2013). Values of 0.01 are considered very small, values of 0.06 moderate, and values of 0.14 large. Deviation contrasts were used to compare the score of each specific group with the combined scores of all other groups. Significant contrasts indicated a statistically significant difference between the focal group and the others. We used a 5% significance level and conducted all analyses using the JASP software.

Researchers who are interested can access the source code and all the results at the following link: https://github.com/HugoKazzi63/mental-health-bertopic.

**Figure 2:**
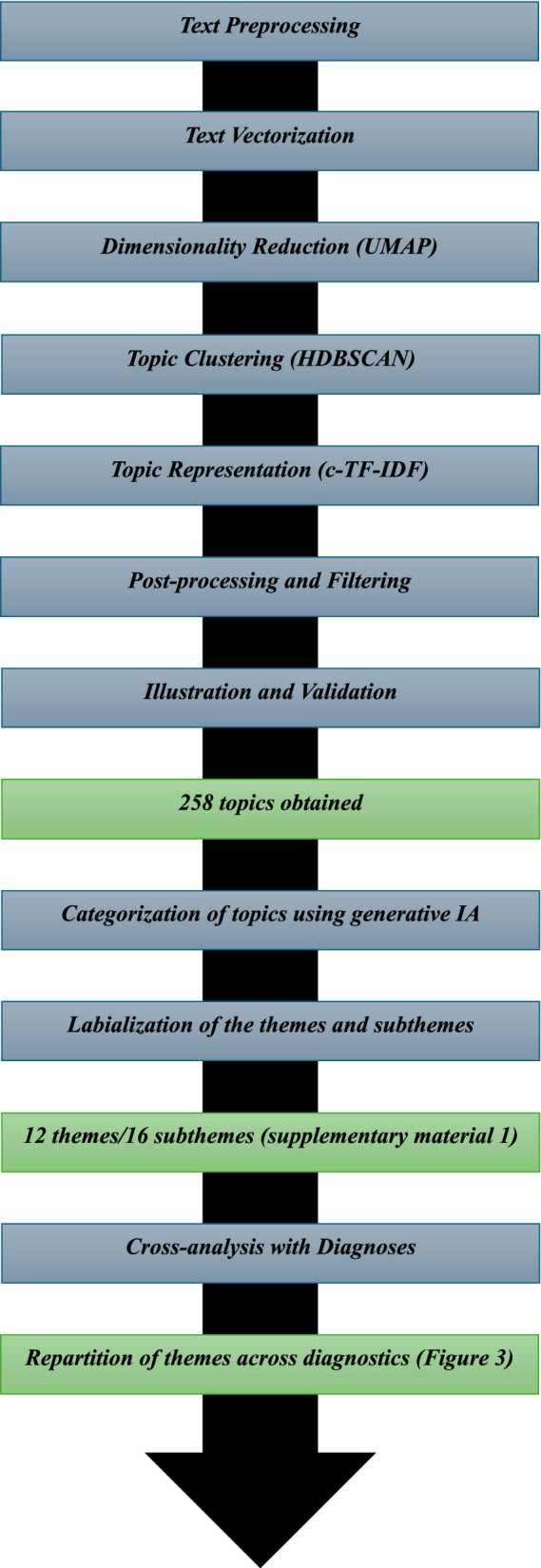
The different steps of the analysis.

All the steps performed for the analysis are available in Figure 2.

## Results

### Automatic thematic analysis

We identified 12 topics in the clinical sample, and between 7 and 12 topics per question in the other samples, leading to a total of 258 topics. Detailed information for each topic, including the original sample, number, title, keywords, summary, prevalence, and typical examples, is available in the Supplementary material 1.

Following automatic categorization, we identified 16 subthemes grouped into 12 broader themes. The first theme was related to emotional and psychological difficulties, encompassing anxiety/stress, depression/mood issues, and other emotional challenges. Participants consistently reported fears about various issues, including social situations, politics, and relationships. They also frequently mentioned depression, self-depreciation, and low energy. Other emotional difficulties included emotions such as anger, nostalgia, and guilt.

Similarly, numerous family and relationship difficulties were mentioned. Participants highlighted the prevalence of loneliness in mental health and the importance of support. They described how familial tensions and conflicts can contribute to emotional distress.

The psychotherapeutic process was frequently discussed. Participants described therapeutic targets such as emotion regulation, interpersonal skills, and self-understanding. They also reported difficulties in accessing mental health professionals and emphasized the need to engage in therapeutic work with a professional.

Among other themes, trauma, grief, and the burden of life events were often cited as sources of suffering. Participants reported the impact of violence, separation, and loss. Issues related to self-identity and low motivation were also commonly mentioned, with individuals struggling with poor self-esteem and a lack of motivation. Many expressed a desire for change, personal growth, and greater autonomy. Work-related stress for professionals and academic pressure for students were also highlighted.

Environmental challenges were described, with participants reporting the impact of financial, social, and physical health factors on their wellbeing. Behavioral difficulties such as addiction and disordered eating were also mentioned. Finally, two topics were related to existential and identity-related questioning, with participants reflecting on the meaning of daily life.

In Table 2, we present each theme and subtheme, along with the number of topics associated with them, including up to five examples to illustrate each. We also indicate the type of phenomena according to Kinderman’s model. Each topic with its corresponding theme is available in Supplementary material.

**Table 2:** Categorization of topics generated through the data.

Theme	Subtheme	Example of topics	Number of topic in the theme/subtheme	Type of phenomenon according to Kinderman’s model
Emotional and psychological difficulties			75	Symptoms
Emotional and psychological difficulties	Anxiety and stress	Anxiety; insomnia; social anxiety and avoidance in public; abandonment and fear of loss; procrastination	43	Symptoms
Emotional and psychological difficulties	Depression and mood disorders	Pessimism about the future; suicidal ideation and self-harm; dark thoughts and mood swings; apathy, energy loss; sadness, anger, and emotional withdrawal	17	Symptoms
Emotional and psychological difficulties	Other emotional difficulties	Emotional vulnerability and narcissistic failure; emotional detachment and rumination; nostalgia and regret; guilt, intrusive thoughts, and obsession; overthinking and rumination	15	Symptoms
Family and social relationships			63	
Family and social relationships	Loneliness and need for support	Lack of engagement and support; violence, social criticism, and isolation; toxic relationships and social isolation; moral support and open dialogue; difficulties with authority and lack of support	27	Social factors
Family and social relationships	Family conflicts and dynamics	Social and family context; divorce, adolescence, and family disruption; couple, housing, and isolation issues; family tensions and social withdrawal; lack of empathy and marital misunderstanding	23	Social factors
Family and social relationships	Other relational difficulties	Lack of communication and institutional dynamics; dependency and difficulty saying no; sibling rivalry and comparison; feeling persecuted and unrecognized; lack of decisiveness and communication	13	Social factors
Therapeutic process			23	
Therapeutic process	Therapeutic techniques and target	Situational analysis and anchoring; emotion regulation and interpersonal skills; strategies and psychoeducation; cognitive restructuring and emotion identification; schema therapy and cognitive restructuring	13	Psychological process
Therapeutic process	Access and effectiveness	No therapy or depends on patient; psychotherapy access and financial barriers; therapeutic alliance and adherence; no ongoing therapy; essential mental health follow-up	10	Psychological process
Trauma and grief	Traumatic events and adaptation	Psychological and emotional aggression; life transitions and environmental change; mockery, judgment, and impostor syndrome; family violence, neglect, and incest; child abuse, violence, and family trauma	23	Circumstantial factors
Self and identity	Motivation and self-perception	Self-confidence and acceptance; emotional improvement and self-confidence; self-esteem and leisure; motivation and engagement; self-confidence and limiting beliefs	20	Psychological process
Desire of better future	Wishing a change	Control over future and personal fulfillment; more balanced activities and time management; independence and autonomy; better organization and self-reflection; healthier; safe environment and reduced expectations relationships and affection; progress in diagnosis and positivity	13	Psychological process
Work and academic pressure	Professional stress and expectations	Time pressure and organizational difficulty; professional failure and ridicule; unemployment, time, and lack of understanding; work routine and lack of response; work-related undervaluation and demotivation	12	Circumstantial factors
Environmental limitation	Money, physical health and social problems limitating change and wellbeing	Stigma and lack of understanding; lack of time, resources, and psychological barriers; physical and neurological health issues; financial, relational, and cognitive difficulties; administrative and diagnostic difficulties	7	Circumstantial factors
Other	Uncategorized	Fatalism and limited change; health challenges and flourishing; skin picking and self-image issues; bodily fatigue and questioning; medication, self-management, and OCD	7	Symptoms
Behavioral and physical health	Addictive and eating behaviors	Emotion and eating regulation; addictive and compulsive behaviors; binge behaviors and escapism; health, diet, and environment; understanding addiction and acceptance	6	Symptoms
Behavioral problems	Agressivity and harmful behavior	Impulsivity, aggression, and emotional reactivity; insomnia and self-harm impulses; social withdrawal and aggressive outbursts; verbal and physical aggression	4	Symptoms
Existential and identity issues	Meaning and fulfillment	Boredom, social withdrawal, and lack of meaning; lack of meaning and constant questioning	2	Symptoms

### Prevalence of topics across diagnoses

We examined the prevalence of different themes and subthemes across various diagnoses. Overall, we did not find any single topic to be uniquely associated with a specific diagnosis. While certain topics were more commonly found among patients with particular diagnoses, topic affiliation was generally distributed across multiple diagnostic categories.

At the descriptive level, the themes of behavioral and physical health, desire for a better future, emotional and psychological difficulties, family and social relationships, the therapeutic process, and trauma and grief showed comparable prevalence across all diagnoses. Other themes were also generally present across all diagnoses, but with some variations. For instance, behavioral problems were more prevalent among participants with addiction disorders; existential and identity-related concerns were more frequent among participants with eating disorders; and work- and academic-related pressure was less common among participants with bipolar disorder.

At the inferential level, the ANOVA did not show a significant effect of diagnosis (*F* = 1.51, *p* = 0.13, η^2^ < 0.01) or theme (*F* = 1.80, *p* = 0.08, η^2^ < 0.01). Similarly, the interaction effect was not significant (*F* = 1.28, *p* = 0.06, η^2^ < 0.01). Taken together, these results indicate that the degree of affiliation with the different themes did not vary significantly across diagnoses, suggesting that all themes were similarly represented in each diagnostic group.

**Figure 3:**
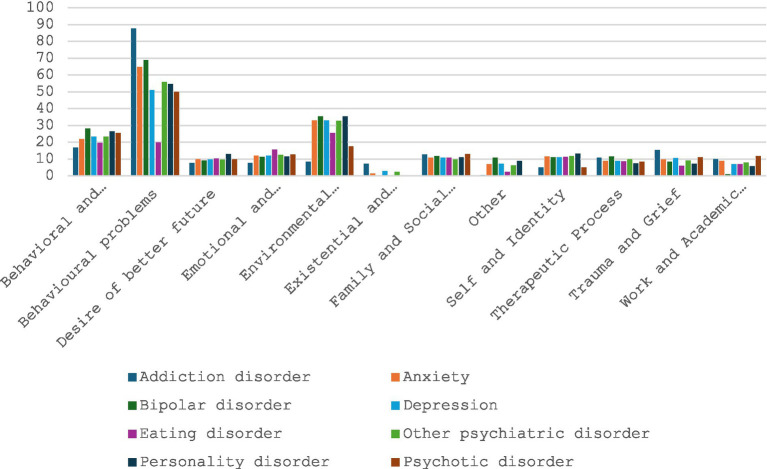
Repartition of the different thematic depending on the diagnostics.

Figure 3 presents the mean level of affiliation for each theme according to participants’ diagnoses. In other words, it shows the extent to which participants with each diagnosis mentioned the different themes in their narratives.

## Discussion

The aim of this research was to explore transdiagnostic symptoms, processes, and distal factors involved in mental health, drawing on experiential knowledge from various populations. Specifically, we sought (i) to identify the different phenomena involved and (ii) to demonstrate their transdiagnostic presence across multiple disorders.

In line with these objectives, we generated multiple topics from participants’ narratives and grouped them into 12 overarching themes.

Following Kinderman (2005) model, we first identified distal factors within the narratives. Participants mentioned circumstantial elements such as traumatic experiences (e.g., violence, bullying) and bereavement. They also described the negative impact of environmental pressures, including financial stress, physical health problems, and lack of time or resources. In addition, they reported the effect of work or academic pressure on wellbeing. These findings are consistent with the scientific literature. For example, previous meta-analyses have documented the association between traumatic experiences and mental health (Hogg et al., 2023). Similarly, income inequality (Patel et al., 2018) and work or academic pressure have been identified as risk factors for mental health problems (Niedhammer et al., 2021; Steare et al., 2023). Relationship difficulties also emerged in our data, aligning with prior research based on traditional quantitative approaches (Lindert et al., 2025).

At the level of psychological processes, we identified the role of self-perception and motivation. Participants emphasized self-esteem, self-confidence, and negative self-evaluations. This is highly consistent with research highlighting the importance of self-esteem across various disorders (e.g., Henriksen et al., 2017; Krauss et al., 2023). Other processes emerged within the theme related to therapeutic mechanisms, such as emotional regulation, cognitive schemas, and attachment difficulties. This aligns with previous models emphasizing the role of emotional dysregulation in many psychological problems (e.g., Cuthbert, 2021; Philippot et al., 2015; Vancappel et al., 2024).

Regarding transdiagnostic symptoms, participants frequently described emotional difficulties, including anxiety, depression, low mood, anger, and related problems. This is congruent with Barlow’s conceptualization, which proposes understanding most psychological disorders as forms of emotional disturbance (Barlow, 2011). Similarly, Goldberg and Hillier (1979) proposed that emotional distress should be viewed broadly as a symptom present in many clinical conditions. Behavioral problems, such as addiction or disordered eating, were also mentioned across samples.

Consistent with our second objective, we found that distal factors, psychological processes, and symptoms occurred in comparable proportions across most diagnoses. This tends to support Kinderman’s, (2005) model.

### Implications

Most previous research has focused primarily on identifying transdiagnostic processes, while giving less attention to circumstantial factors and symptoms. Our findings suggest that not only psychological processes but also symptoms and distal factors can be shared across disorders. Some of these elements—such as relational difficulties—have received limited attention and warrant deeper exploration in future studies.

Methodologically, this research illustrates how AI-based techniques can be used to analyze psychological data, offering new possibilities for qualitative research. In particular, these methods enable the examination of large volumes of narratives. Further studies should refine this approach to improve its accuracy and applicability in psychology and psychiatry.

### Limitations

This study has several limitations. First, we relied on self-reported data. Although we included diverse samples, participants needed to be aware of the psychological mechanisms under investigation to describe them accurately. The sample was also unbalanced, with a larger proportion of participants from the general population. Moreover, the narratives were often brief; semi-structured interviews would have provided richer data to better support AI-based analyses. The questions posed to the clinical sample differed slightly, as they were derived from retrospective data, and diagnostic categories were broad; more precise diagnoses might have improved accuracy. Finally, Natural Language Processing has intrinsic limitations: it relies on statistical analysis of word frequencies and does not fully capture the meaning of narratives. For instance, metaphors may generate artificial relationships between words. Researchers should therefore use caution when interpreting results from such techniques. Finally, we used GPT-4.0 to both classify and describe the 258 topics into themes and subthemes. This approach may be subject to criticism, and future research should compare it with a human-based classification process.

## Conclusion

This study had two main aims. First, we sought to explore the phenomena that may be involved in psychopathology, following Kinderman (2005) general conceptualization. This includes distal factors (biological, circumstantial, and social), proximal factors (psychological processes), and symptomatology. The second aim of this paper was to strengthen the idea that similar symptoms, processes, and distal factors are involved across different diagnoses, using new data-analysis and natural language processing tools. We recruited four samples of participants: patients provided first-hand accounts of lived experience, relatives contributed insights into interpersonal dynamics, clinicians brought professional expertise, and members of the general population offered a normative reference point. In total, 1,500 responses were collected.

Participants responded to open-ended questions exploring multiple phenomena that can generate psychological suffering (e.g., emotions, thoughts, social situations). We used natural language processing and AI-based analyses to extract the main topics in the responses. We identified 258 topics, which were organized into 12 overarching themes. The most prominent topics concerned Emotional and Psychological Difficulties (e.g., anxiety, avoidance, depression), Family and Social Relationships (e.g., isolation, lack of support), and Therapeutic Processes (e.g., cognitive restructuring, emotion-regulation training).

We examined the prevalence of the different themes and subthemes across various diagnoses. Overall, we did not find any single topic to be uniquely associated with a specific diagnosis. Topic distribution generally spanned multiple diagnostic categories.

Further research should replicate this study by increasing the variety of algorithms used for topic extraction and by analyzing longer narrative segments, such as those obtained from semi-structured interviews. Finally, quantitative measures should be developed to assess the different phenomena identified in this study and to compare their prevalence across psychiatric disorders. Future research should also consider using natural language processing to create decision-support systems that help clinicians evaluate and detect the psychological phenomena presented by their patients based on narratives and textual information.

## Data Availability

The raw data supporting the conclusions of this article will be made available by the authors, without undue reservation.
